# Regulation of Embryonic Cell Adhesion by the Prion Protein

**DOI:** 10.1371/journal.pbio.1000055

**Published:** 2009-03-10

**Authors:** Edward Málaga-Trillo, Gonzalo P Solis, Yvonne Schrock, Corinna Geiss, Lydia Luncz, Venus Thomanetz, Claudia A. O Stuermer

**Affiliations:** Department of Biology, University of Konstanz, Konstanz, Germany; Scripps Research Institute, United States of America

## Abstract

Prion proteins (PrPs) are key players in fatal neurodegenerative disorders, yet their physiological functions remain unclear, as PrP knockout mice develop rather normally. We report a strong PrP loss-of-function phenotype in zebrafish embryos, characterized by the loss of embryonic cell adhesion and arrested gastrulation. Zebrafish and mouse PrP mRNAs can partially rescue this knockdown phenotype, indicating conserved PrP functions. Using zebrafish, mouse, and *Drosophila* cells, we show that PrP: (1) mediates Ca^+2^-independent homophilic cell adhesion and signaling; and (2) modulates Ca^+2^-dependent cell adhesion by regulating the delivery of E-cadherin to the plasma membrane. In vivo time-lapse analyses reveal that the arrested gastrulation in PrP knockdown embryos is due to deficient morphogenetic cell movements, which rely on E-cadherin–based adhesion. Cell-transplantation experiments indicate that the regulation of embryonic cell adhesion by PrP is cell-autonomous. Moreover, we find that the local accumulation of PrP at cell contact sites is concomitant with the activation of Src-related kinases, the recruitment of reggie/flotillin microdomains, and the reorganization of the actin cytoskeleton, consistent with a role of PrP in the modulation of cell adhesion via signaling. Altogether, our data uncover evolutionarily conserved roles of PrP in cell communication, which ultimately impinge on the stability of adherens cell junctions during embryonic development.

## Author Summary

Unlike conventional pathogens, prions are infectious particles devoid of nucleic acids and composed entirely of a misfolded host protein, PrP. It is widely assumed that the neurodegeneration observed in prion disorders may be related to an aberrant function of PrP in the misfolded state. However, the normal physiological function of PrP remains poorly understood, mainly owing to the absence of clear phenotypes in mice lacking PrP. Here, we show that when PrP is depleted in zebrafish embryos, dramatic phenotypes ensue, severely affecting the development of early and late (neural) structures. We examined the mechanisms responsible for some of these defects, and found that fish and mammalian PrPs play conserved roles in cell–cell communication, by directly mediating cell adhesion and by triggering cellular signals that further modulate the function of other adhesion molecules. In the early zebrafish embryo, these activities control not only tissue integrity and cell morphology, but also the complex cellular movements that give rise to germ layers. This study describes—to our knowledge—the first known in vivo function of PrP and its molecular cellular basis, which may provide helpful insights into the role of PrP in the adult brain and its proposed connections to prion-induced neurotoxicity.

## Abstract 3

Knockdown experiments in zebrafish embryos reveal that prion proteins, otherwise known for their involvement in lethal neurodegenerative disease, play important roles in cell-cell adhesion and signaling during embryonic development.

## Introduction

The prion protein (PrP) is a membrane-anchored glycoprotein, best known for its unique ability to undergo structural conversion from a normal “cellular” isoform (PrP^C^) into a pathogenic conformer known as “scrapie” (PrP^Sc^) [1]. The accumulation of scrapie aggregates—prions—in the brain is a distinctive feature of transmissible spongiform encephalopathies, a group of lethal neurodegenerative diseases that include Kuru and Creutzfeldt-Jacob disease in humans, scrapie in sheep, and mad cow disease in cattle [2]. While much has been learned about the pathogenic properties of PrP, its normal physiological role remains elusive [3,4]. We previously identified PrP orthologs in fish and proposed that the conservation of their protein domain architecture reflects the maintenance of an ancient and important biological role of PrP across vertebrates [5].

Although PrP is widely expressed in mouse embryos [6], PrP knockout mice are surprisingly viable and show no major physical or behavioral abnormalities [7]. For the last 17 years, this lack of in vivo phenotypes has precluded PrP from genetic functional analysis, raising the intriguing question of whether its unknown physiological function is necessary or dispensable for the organism, and also whether prion neurotoxicity may be a consequence of PrP mis- or loss-of-function. So far, diverse roles have been proposed for PrP^C^, including signal transduction [8], cell adhesion and protection from apoptosis and oxidative stress [4], as well as neurogenesis [9,10], axonal growth [11], hematopoietic stem cell self-renewal [12], and lymphocyte activation [13,14]. However, these potential PrP functions do not seem to share a common molecular basis, and their in vivo relevance remains to be clarified.

Here, we show that early down-regulation of PrP impairs cell adhesion in the zebrafish embryo, disrupting morphogenetic cell movements and ultimately causing developmental arrest. Using aggregation assays, we established that PrP subserves complex roles in both Ca^+2^-independent and Ca^+2^-dependent cell adhesion. Our analyses of morphant embryonic cells revealed that PrP is required for the proper membrane localization of E-cadherin adhesion complexes. We also carried out experiments in *Drosophila* S2 cells to demonstrate that PrP itself induces homophilic cell adhesion, and that its accumulation at cell contacts leads to the recruitment of microdomain-associated proteins, eliciting signal transduction and rearrangement of the actin cytoskeleton. Finally, we found that the roles of PrP in cell adhesion and signaling are conserved across vertebrate classes, and that PrP interactions can take place even between mouse and fish orthologs. Our results contribute novel molecular and cellular aspects of PrP function in vitro and in vivo, which may be of relevance to understanding its long-sought physiological roles in the mammalian brain, as well as the potential link between PrP loss-of-function and prion-induced neurodegeneration.

## Results

### PrP Expression during Zebrafish Development

**Figure 1:**
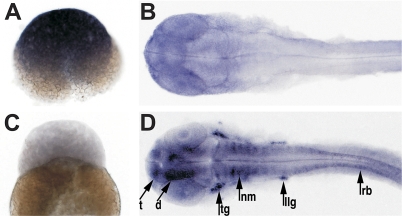
Differential Expression of *PrP* Genes in Zebrafish Embryos The developmental expression patterns of *PrP-1* and *-2* were examined by whole mount in situ RNA hybridization using gene-specific probes. At mid-blastula stages (2.5 hpf, [A and C]), *PrP-1* is ubiquitously transcribed at high levels and *PrP-2* is not detectable. At pharyngula stages (30 hpf, [B and D]), low levels of *PrP-1* transcripts appear restricted to the forebrain and eyes, while *PrP-2* becomes strongly transcribed in defined neural structures. (A and C) show lateral views; (B and D) show dorsal views. d, diencephalon; llg, lateral line ganglion; nm, neuromeres; rb, Rohon-Beard sensory neurons; t, telencephalon; tg, trigeminal ganglion.

Because zebrafish contain duplicated *PrP* genes [5], *PrP-1* and *-2*, we assessed their degree of functional overlap by examining their patterns of embryonic expression. RNA in situ hybridization shows strong and ubiquitous distribution of maternal *PrP-1* transcripts at early midblastula stages (2.5 h postfertilization [hpf], Figure 1A); this is followed by a sharp decrease after gastrulation to reach minimal levels in the forebrain and eyes at the pharyngula stage (30 hpf, Figure 1B). In contrast, *PrP-2* transcripts remain undetectable from 2.5 hpf (Figure 1C) until somitogenesis but reach high levels by 30 hpf, especially in the brain (telencephalon and diencephalon), as well as in discrete neuronal populations of the central nervous system (CNS) (trigeminal ganglia and neuromeres) and peripheral nervous system (PNS) (lateral line ganglia and Rohon-Beard neurons) (Figure 1D, arrows). These complementary expression patterns, which were not evident in previous studies [15], indicate that fish *PrP-1* and *-2* carry out specialized roles during distinct developmental stages: *PrP-1* transcripts are abundant at early stages characterized by active cell division and migration in the entire embryo, whereas *PrP-2* is specifically up-regulated later in the developing nervous system, particularly in neurogenic placodes.

### PrP Loss-of-Function Phenotypes

**Figure 2:**
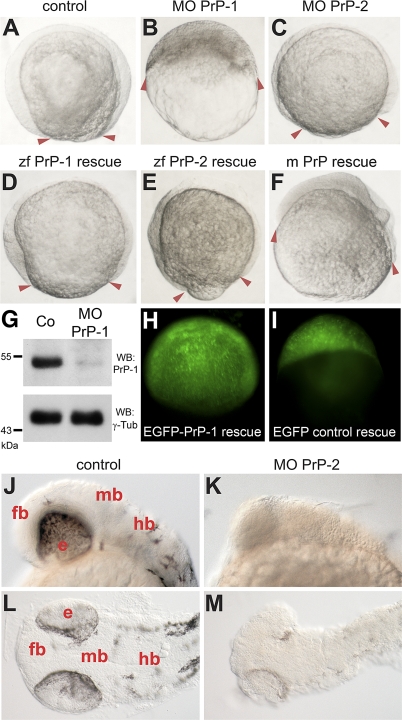
PrP Morpholino Knockdown and RNA Rescue in Zebrafish Embryos (A–F) Embryos were microinjected at one- to four-cell stages and photographed at 9 hpf. (A) Control embryos reach approximately 90% epiboly, as evidenced by the normal progression of the blastodermal margin (red arrowheads). (B) Epiboly in PrP-1 morphant embryos is severely impaired, and they remain arrested. (C) No appreciable morphological defects are seen in PrP-2 morphant embryos. In rescue experiments, the morpholino (MO) PrP-1 phenotype is reverted to different extents by microinjection of *PrP-1* (D), *PrP-2* (E), or mouse *PrP* (F) mRNAs. m, mouse; zf, zebrafish. (G) Western blot (WB) analysis of 6-hpf embryo cell extracts shows that PrP-1 expression (∼53-kDa band) is effectively suppressed by morpholino injection. Co, control. (H and I) Embryos expressing the *EGFP-PrP-1* fusion mRNA can overcome the MO PrP-1 arrest, normally reaching 75% epiboly at 8 hpf (H). In contrast, embryos expressing the corresponding control EGFP fusion mRNA remain arrested (I). (J–M) At the prim-5 (24 hpf) stage, PrP-2 morphant embryos (K and M) show severe malformations in the head region, compared to control embryos (J and L). (J and K), lateral views; (L and M), dorsal views. e, eye; fb, forebrain; hb, hindbrain; mb, hindbrain.

The developmental expression patterns of *PrP-1* and *-2* indicated that they perform early ubiquitous and late neural embryonic roles, respectively. To test this prediction, we blocked the translation of each gene by microinjecting antisense morpholinos into one- to four-cell stage embryos. Such knockdown of PrP-1 produced a strong early phenotype characterized by the failure to carry out gastrulation beyond 6 hpf (shield stage), resulting in a large proportion of arrested embryos at 9 hpf (∼95%, *n* = 200) (Figure 2B) compared to control embryos (5%, *n* = 200) (Figure 2A, Table S1). Western blot analysis of 6-hpf embryos confirmed that PrP-1 expression levels were effectively suppressed by morpholino microinjection (Figure 2G). On the other hand, PrP-2 knockdown embryos showed normal gastrulation (Figure 2C) and survived into early larval stages (≥7 d postfertilization [dpf]), but presented morphological defects in the head region, particularly malformed brains and eyes (∼65%, *n* = 200) (Figure 2K and 2M). These early and late phenotypes correlate with the developmental expression patterns of *PrP-1* and *-2*, respectively. Thus, the gastrulation arrest in PrP-1 morphant embryos reveals that this protein is essential for epiboly (the spreading of the blastodisc from the animal to the vegetal pole), whereas the malformations observed in PrP-2 morphant embryos are consistent with a specific role of this protein in neural differentiation and brain morphogenesis.

**Figure 3:**
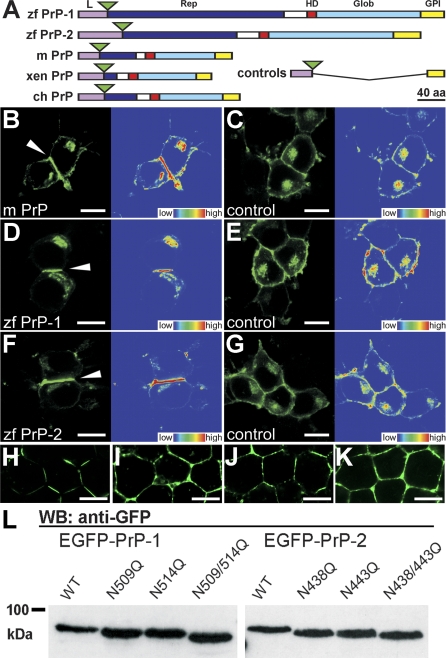
Expression of EGFP-Tagged PrPs in N2a Cells and in Zebrafish Embryos (A) EGFP fluorescent constructs used in this study: the major domains of zebrafish (zf PrP-1 and -2), mouse (m PrP), *Xenopus* (xen PrP), and chick (ch PrP) prion proteins are indicated: leader peptide (L) in violet, repetitive region (Rep) in blue, hydrophobic domain (HD) in red, globular domain (Glob) in light blue, and GPI-anchored peptide (GPI) in yellow. Fluorescence tags are represented as green triangles; control constructs (controls) lack PrP cores (Rep, HD, and Glob); domains are shown as previously defined [5]. (B, D, and F) N2a cells transfected with mouse PrP (B), zebrafish PrP-1 (D) and PrP-2 (F) constructs show local accumulation of the fusion proteins at cell–cell contacts (white arrowheads and fluorescence profiles, right). (C, E, and G) The corresponding control EGFP fusion constructs are evenly distributed along the plasma membrane. (H–K) For visualization of PrP expression in zebrafish deep cells, embryos were microinjected with zebrafish PrP-1 (H), PrP-2 (I), mouse PrP (J), and PrP-1 (control [K]) EGFP fusion RNAs and analyzed at the sphere stage (4 hpf). (L) Predicted N-glycosylation sites of zf PrP-1 and -2 were confirmed by Western blot (WB) analysis (anti-GFP monoclonal antibody) of extracts from N2a cells transfected with the constructs indicated above each lane. WT, wild type. (B–G) show EGFP fluorescence (left), and total fluorescence profiles (right); (H–J) show EGFP fluorescence and Nomarski overlays. Scale bars in (B–G) indicate 10 μm; scale bars in (H–J) indicate 5 μm.

Given their shared protein domain composition, PrP-1 and -2 are likely to have similar biological activities, despite their distinct amino acid sequences and developmental expression patterns. To examine the degree of functional relatedness between zebrafish (and mouse) PrPs, we tested their ability to rescue the PrP-1 loss-of-function phenotype. Embryos were coinjected with various combinations of *PrP-1* morpholino and in vitro synthesized *PrP* mRNAs lacking the morpholino binding sites. As expected, the severity of the PrP-1 early phenotype could be rescued with *PrP-1* mRNA (21% of arrested embryos, *n* = 200) (Figure 2D): coinjected embryos overcame the gastrulation arrest and survived into larval stages. Remarkably, partial rescue of the PrP-1 knockdown was also observed upon coinjection of *PrP-2* (38% of arrested embryos, *n* = 200) (Figure 2E) and even mouse *PrP* mRNAs (47% of arrested embryos, *n* = 200) (Figure 2F). Differences in rescue efficiency between these mRNAs were also seen in the variation of the degree of epiboly attained at 9 hpf: while control embryos and PrP-1 morphants reached about 90% (Figure 2A, arrowheads) and 50% epiboly (Figure 2B, arrowheads), respectively, the zebrafish (Figure 2D and 2E, arrowheads) and mouse PrP (Figure 2F, arrowheads) rescues attained about 90% and 70% epiboly at this time, respectively (Table S1). Rescues using the corresponding *EGFP-PrP* fusion mRNAs (Figure 3A) produced similar results (Figure 2H and Table S2) and allowed us to visualize ubiquitous expression of the rescuing fusion proteins (Figure 2H and 2I). Furthermore, control mRNAs coding for only the PrP leader and GPI-anchor peptides (Figure 3A, controls) could not revert the PrP-1 phenotype (87.5% of arrested embryos, Figure 2I and Table S2), confirming that the rescue ability depends on the presence of the PrP cores (repetitive, hydrophobic, and globular domains). In contrast, the PrP-2 phenotype could not be rescued due to the technical limitation of having to inject the mRNAs at the one- to four-cell stages: rescuing mRNAs were inevitably expressed at blastula stages, causing early ectopic overexpression before the endogenous *PrP-2* could actually be transcribed and therefore targeted by the morpholino. Interestingly, ectopic (ubiquitous) overexpression of zebrafish or mouse *PrP* mRNAs produced similar morphological phenotypes (Figure S1): asymmetric epiboly and severe defects in eye and brain morphology. Thus, although not identical, PrP down-regulation and overexpression phenotypes converge at the same developmental processes (gastrulation and neural development) where a basic cellular function shared by fish and mouse PrPs is required.

### Conserved Cellular Properties of PrPs

To gain preliminary insight into the cellular mechanisms responsible for the phenotypes observed, we analyzed the heterologous expression of zebrafish PrPs in mouse neuroblastoma 2a (N2a) cells, a neuronal cell line routinely used to study the functional and pathogenic properties of PrP. To overcome the lack of anti–zebrafish-PrP antibodies suited for immunofluorescence, we used EGFP-PrP fusion constructs (Figure 3A). In these experiments, expression of zebrafish and mouse EGFP-PrP constructs in N2a cells led to strong protein accumulation at cell contacts (Figure 3B, 3D, and 3F, arrowheads and fluorescence profiles). This phenomenon was not observed upon surface expression of control EGFP constructs (Figure 3C, 3E, and 3G), indicating that the PrP leader and GPI-anchor peptides are sufficient for protein targeting and attachment to the cell membrane, but that the accumulation at cell contacts is dependent on the presence of the PrP cores. Moreover, PrP accumulation was observed only when both cells forming the contact expressed the PrP construct (Figure S2A), suggesting that PrPs might engage in homophilic *trans*-interactions. Interestingly, while mouse PrP and zebrafish PrP-2 were observed along the entire cell membrane (Figure 3B and 3F), PrP-1 localized almost exclusively at cell contacts (Figure 3D), suggesting a contact-dependent regulation of PrP-1 membrane positioning. PrP accumulation at N2a cell contacts could also be observed by immunostaining endogenous PrP with a specific monoclonal antibody (Figure S2B), as well as by using DsRed-monomer constructs (Figure S2C–S2H), and in HeLa cells (data not shown). To examine zebrafish and mouse PrP expression in vivo, we microinjected the corresponding *EGFP-PrP* mRNAs into zebrafish embryos. The localization patterns observed at 6 hpf (Figure 3H–3K) were consistent with those seen in N2a cells, including the relatively homogeneous membrane distribution of PrP-2 and mouse PrP (Figure 3I and 3J), the local accumulation of PrP-1 in patches at cell contacts (Figure 3H), and even the loss of discrete accumulation by a control construct lacking the PrP-1 core (Figure 3K). Similar to their mammalian counterparts, attachment of zebrafish PrPs to N2a cell membranes via GPI-anchors was greatly reduced by PI-PLC treatment (Figure S2I and S2J); likewise, N-glycosylation could be demonstrated by PNGase F digestion (see Western blots in Figure S2K). We also generated mutant constructs for PrP-1 and PrP-2 in which the putative N-glycosylation residues were point mutated to glutamine. Western blot analysis of these constructs confirmed that, like mammalian PrPs, zebrafish PrP-1 and -2 are mono- and di-glycosylated at the expected residues (Figure 3L). These experiments strengthen the concept that the cellular function of PrP is conserved between fish and mammals.

### PrP-1–Mediated Embryonic Cell Adhesion

**Figure 4:**
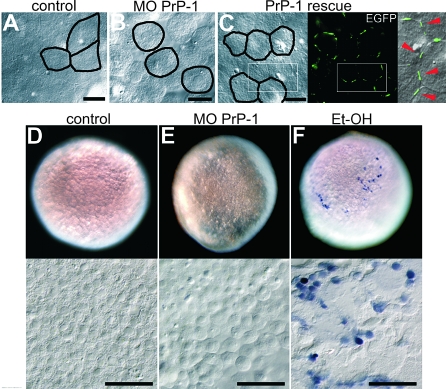
Effect of PrP-1 Knockdown in Embryonic Cell Adhesion Differences in tissue compactness between deep cells of control, morphant, and rescued embryos were evaluated at the shield stage (6 hpf ). (A) Control embryos exhibit normal tissue compactness and polygonal cell shapes. (B) Reduced cell adhesion and rounded cells are evident in PrP-1 morphant (MO) embryos. (C) Local accumulation of EGFP-PrP-1 at cell contacts (see red arrowheads in detailed overlay view of framed region, right) reverts these effects in rescued embryos. Cell outlines were digitally redrawn to help visualization of cell shape. (D–F) The loss of embryonic cell adhesion is not related to cell-death, as whole-mount TUNEL stainings of control (D), morphant (E), and ethanol-treated embryos (F) show apoptotic cells (blue staining) only in (F). Scale bars in (A–C) indicate 10 μm; scale bars in (D–F) indicate 50 μm.

The specific accumulation of PrP at cell contacts suggested that the zebrafish PrP phenotypes could be explained by defects in cell–cell communication. Given the relative simplicity and ease of manipulation of the early zebrafish embryo, we focused our analysis on the cellular and molecular characterization of the PrP-1 phenotype. Morphological examination of PrP-1 knockdown embryos at 6 hpf revealed that the developmental arrest was preceded by a marked decrease in tissue integrity and compactness; as they detached, deep cells in the morphant embryo lost their otherwise polygonal shape and became round (Figure 4A and 4B). The progressive loss of cell adhesion was clearly not a consequence of cell death, as in control embryos, death at this stage usually leads to generalized cell lysis within a few minutes. In contrast, round morphant cells survived at least until 12 hpf. Moreover, TUNEL (terminal deoxynucleotidyl transferase-mediated dUTP nick-end labeling) and DAPI stainings of 6-hpf PrP-1 morphant embryos showed no signs of apoptotic DNA fragmentation (Figures 4D–4F and S3A–S3D). Thus, the loss of embryonic cell adhesion is a specific effect of PrP-1 knockdown, which can be rescued by the local accumulation of exogenous PrPs at cell contacts sites (Figure 4C, arrowheads).

**Figure 5:**
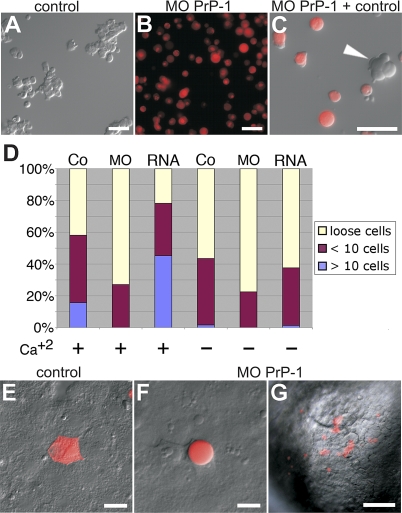
Aggregation and Cell Transplantation Assays Using PrP-1 Morphant Blastomeres Shield stage (6 hpf) embryos were dissociated to single cells, the cells were allowed to reaggregate in suspension for 45 min and were then plated out for analysis. (A) Control embryos normally form small and large cell clusters. (B) Aggregation is considerably reduced in PrP-1 morphant cells. (C) When cocultured, PrP-1 morphant cells are generally excluded from control cell aggregates (white arrowhead). (D) Quantitative differences in aggregation potential were observed between PrP-1 morphant, control, and PrP-1 overexpressing cells in the presence and absence of Ca^+2^; the bar graph displays the ratios of loose, small (<10 cells), and large (>10 cells) aggregates in control (Co) and PrP-1 morphant (MO) cells, 45 min after dissociation. (E–G) Cell-autonomy of the PrP-1 defect was tested by embryonic cell transplantation: Labeled control deep cells (E) integrate into the control host tissue and resume polygonal shape. In contrast, PrP-1 morphant deep cells (F and G) fail to establish cell contacts with the control host cells. Adhesive properties were evaluated at 6 hpf, 2 h after transplantation. Scale bars in (A–C, E, and F) indicate 20 μm; scale bar in (G) indicates 100 μm.

To quantitatively assess the cell adhesion defect in PrP-1 knockdown embryos, we prepared primary cultures of dissociated zebrafish blastomeres (single-cell suspensions of 6-hpf embryos) and tested their reaggregation potential in the presence of Ca^+2^. After 45 min in suspension, control cells formed cell aggregates with an average size of 4.5 ± 0.2 cells/aggregate (maximum size = 29 cells/aggregate), whereas PrP-1 morphant cells formed significantly smaller aggregates (*p* < 0.001) with an average size of 2.7 ± 0.1 cells/aggregate (maximum size = 9 cells/aggregate) (Figure 5A and 5B). Moreover, when dissociated control and morphant cells were cocultured, compact aggregates of control cells formed rapidly (within 5 min), from which loose morphant cells were often excluded (Figure 5C, arrowhead).

During gastrulation, cell adhesion is dynamically maintained by cadherin homophilic interactions [16]. This raised the possibility that the PrP-1 knockdown phenotype could be due—at least partly—to the misregulation of cadherin function, which is Ca^+2^ dependent [17]. To test this hypothesis, we performed aggregation assays with dissociated control and PrP-1 morphant cells in the presence and absence of Ca^+2^. When the assay was performed in the Ca^+2^-containing medium, PrP-1 morphant cells underwent a significant decrease in the number of large (>10 cells) and small (<10 cells) aggregates (100%, *p* = 0.0004; and 36% reduction, *p* = 0.003, respectively), compared to control cells (Figure 5D, + bars). The same relative effect was observed when the assay was performed in Ca^+2^-free medium, indicating that PrP-1 is required for the formation of Ca^+2^-independent cell clusters, mostly of small size (Figure 5D, − bars). On the other hand, aggregation of PrP-1 morphant cells in the presence or absence of Ca^+2^ showed no significant differences, implying that the formation of large clusters is mediated by cadherins and controlled by PrP-1. Interestingly, when the assay was performed with PrP-1 overexpressing cells in the presence of Ca^+2^, a dramatic increase in the number of large clusters was recorded (200% increase, *p* = 4 × 10^−7^, Figure 5D, + bars); in the absence of Ca^+2^, PrP-1 overexpressing cells virtually formed no large clusters (as expected), and the number of small clusters was larger than that of PrP-1 knockdown cells, and comparable to that of control cells (Figure 5D, − bars). These results are consistent with a complex role of PrP-1 in the maintenance of embryonic cell adhesion via cell-autonomous interactions at the plasma membrane. To test for cell autonomy, we transplanted small groups of deep cells from 4-hpf blastulae treated with *PrP-1* and control morpholinos into 4-hpf control blastulae (Figure 5G). After 2 h, control cells established normal cell contacts within the control host embryo and acquired polygonal morphology (10 out of 12 experiments, Figure 5E). In contrast, PrP-1 morphant cells remained round and loose within the control host embryo (10 out of 10 experiments, Figure 5F). Thus, the adhesion defect in PrP-1 morphant cells is cell-autonomous and cannot be corrected by the cellular environment of the control host embryo. Unfortunately, transplantation of control cells into a morphant host was not informative because PrP-1 morphant embryos did not withstand manipulation.

### PrP-1 and the Regulation of E-cadherin–Mediated Cell Adhesion

**Figure 6:**
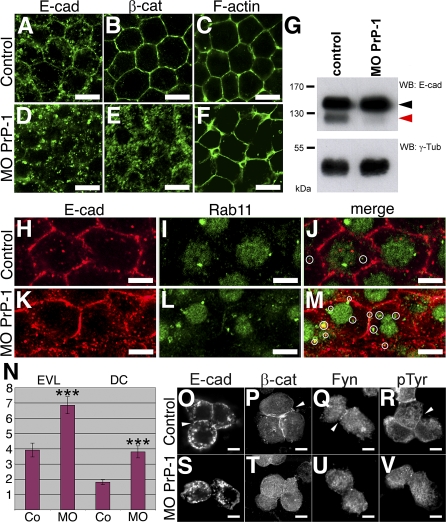
Regulation of Cadherin-Mediated Cell Adhesion by PrP-1 Differences in the subcellular distribution of adherens junction components between control and PrP-1 morphant (MO) embryos were evaluated in the deep cell layer at the shield stage (6 hpf). (A–F) The normal membrane localization of E-cadherin (E-cad [A]), β-catenin (β-cat [B]), and cortical F-actin (C) in control embryos appears disrupted upon PrP-1 knockdown (D–F). (G) Western blot (WB) analysis of embryo cell extracts (6 hpf) reveals an almost complete reduction in the relative levels of mature E-cadherin (120 kDa, red arrowhead), and a slight increase in the levels of its immature form (140 kDa, black arrowhead) upon PrP-1 knockdown. γ-Tub, γ-tubulin. (H–M) Changes in the number of Rab11-positive vesicles containing E-cadherin between control (Co [H–J]) and PrP-1 morphant embryos (K–M) were analyzed by immunostaining. Compared to control embryos, PrP-1 morphant embryos exhibit a higher density of E-cadherin/Rab11 double-positive vesicles in deep cells (white circles in [J and M]). (N) The quantitative difference in the number of E-cadherin/Rab11 colocalizations per cell (*y*-axis) between control (Co) and PrP-1 morphant embryos (MO) was statistically significant for EVL (*p* = 0.0002) and DC (deep cells, *p* = 0.0004); triple asterisks (***) indicate statistical significance at *p* < 0.001. Error bars indicate SEM. (O–V) Accumulation of E-cadherin (O), β-catenin (P), Fyn tyrosine kinase (Q), and phosphotyrosine staining (R) at cell contacts between primary blastomeres derived from control embryos (white arrowheads) is lost in PrP-1 morphant blastomeres (S, T, U, and V). Scale bars in (A–F) indicate 10 μm; scale bars in (H–M and O–V) indicate 5 μm.

Our aggregation assays indicated that PrP-1 can modulate Ca^+2^-dependent cell adhesion in the embryo. Therefore, we investigated whether PrP-1 knockdown would affect the expression and subcellular localization of E-cadherin. Since cadherin homophilic interactions are anchored to the actin cytoskeleton via catenins [18], we also analyzed PrP-1–mediated changes in the distribution of β-catenin and F-actin. Antibody and phalloidin stainings revealed the typical cell surface localization of these molecules in 6-hpf control embryos (Figure 6A–6C). In contrast, E-cadherin and β-catenin appeared largely intracellular in PrP-1 morphant cells, and the distribution of F-actin was disorganized (Figure 6D–6F). This apparent intracellular accumulation of E-cadherin could be due to increased E-cadherin endocytosis and/or degradation, or to deficient trafficking to the plasma membrane. To address these possibilities, we first carried out Western blot analysis on cell extracts from 6-hpf control and PrP-1 morphant embryos. Notably, PrP-1 morphant cells showed an almost complete reduction in the levels of the 120-kDa polypeptide reported to be the active membrane-bound form of E-cadherin [19], as well as a slight increase in the levels of the 140-kDa immature form of E-cadherin [19], thought to be abundantly stored in intracellular compartments [20] (Figure 6G). Previous studies have identified the recycling endosome, with its associated small GTPase Rab11, as an intermediate compartment that regulates post-Golgi trafficking and exocytosis of E-cadherin to the plasma membrane [21]. We reasoned that if the increased intracellular distribution of E-cadherin in PrP-1 morphant cells was due at least in part to deficient delivery of E-cadherin to the plasma membrane, then E-cadherin would be seen to accumulate in Rab11-positive intermediate compartments. Therefore, we analyzed the subcellular distribution of E-cadherin and Rab11 in 6-hpf embryos by double immunostainings, and quantitatively assessed changes in their degree of colocalization upon PrP-1 knockdown. We found that the marked intracellular distribution of E-cadherin in morphant embryos was accompanied by a significant increase (*p* < 0.001) in the number of E-cadherin/Rab11 double-positive vesicles (Figure 6H–6N). These experiments suggest that PrP-1 can modulate the function of E-cadherin by regulating its processing and/or transport from intracellular stores to the plasma membrane.

To verify that PrP-1 can specifically modulate the stability of adhesion complexes at discrete cell–cell contacts (as opposed to within a tissue), we also carried out immunostainings on dissociated blastomeres that had been allowed to reaggregate. Comparison of control and morphant cells showed that the local accumulation of E-cadherin and β-catenin at newly formed cell contacts requires PrP-1 (Figure 6, compare 6S and 6T with 6O and 6P). Moreover, we observed PrP-1–dependent accumulation of Fyn tyrosine kinase and tyrosine phosphorylated proteins at cell contacts (Figure 6, compare 6U and 6V with 6Q and 6R), suggesting that the regulation of E-cadherin localization by PrP-1 involves the activation of Src-related kinases and downstream targets. We asked next whether the regulatory role of PrP-1 over E-cadherin could be further confirmed by showing that the two molecules interact at the genetic level. To test for synergistic interactions, we microinjected embryos with low doses of PrP-1 morpholino, E-cadherin morpholino, or the two morpholinos together, and scored the number of embryos with arrested gastrulation at 6 hpf. Our results show a statistically significant increase (*p* < 0.005, *n* = 3, ∼400 embryos per experiment) in the percentage of arrested embryos for the PrP-1/E-cadherin double knockdown (88.75 ± 1%), compared to the PrP-1 (46.02 ± 0.8%) or to the E-cadherin (38.16 ± 0.46%) single knockdowns, or to the control morpholino (1.29 ± 0.23%). These data strongly suggest that PrP-1 and E-cadherin act synergistically to regulate embryonic cell adhesion.

**Figure 7:**
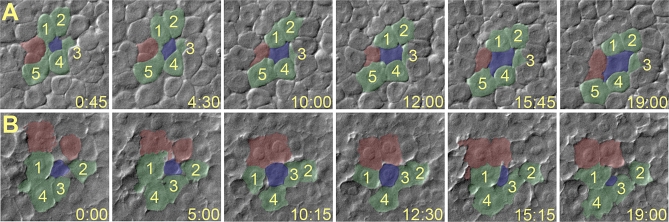
Radial Intercalation in Control and PrP-1 Morphant Embryos Cell movements in lateral regions of the epiblast (8 hpf) were imaged over 20 min using Nomarski optics. (Time in minutes and seconds is shown in the lower-right corner of each panel.) Selected frames from time-lapse video recordings were pseudocolored: intercalating and surrounding cells are shown in blue and green, respectively (after Kane et al. [22]), and cells forming new contacts are shown in red. (A) In control embryos, cells from the interior layer (blue) intercalate among cells in the exterior layer (green) and establish additional cell contacts with other cells (red). (B) In PrP-1 morphant embryos, cells from the interior layer often intercalate and then deintercalate, thereby failing to maintain attachment to cells from the exterior layer (green cells 2 and 4, and red cells).

Kane et al. [22] have demonstrated that E-cadherin mutants fail to carry out epiboly because radial intercalation—a morphogenetic cell movement crucial for the spreading of the blastoderm over the yolk cell—is impaired. In these mutants, cells from the interior layer of the epiblast move normally to the exterior layer but fail to integrate and become restricted, thereby blocking the expansion of the blastoderm. Because E-cadherin adhesion is affected in PrP-1 morphants, we hypothesized that their epibolic arrest could be explained by defective radial intercalation. To confirm this, we carried out time-lapse recordings of cell behavior in the epiblast. In control embryos (Figure 7A), intercalating cells (in blue) entered the exterior layer and flattened out within approximately 20 min, effectively increasing their area. During this process, cells from the exterior layer that were initially in contact with the intercalating cell (in green), or that established new contacts with it (in red), remained in stable contact. In PrP-1 morphant embryos (Figure 7B), intercalating cells entered the exterior layer and increased their area, but did not completely flatten out: after approximately 10 min, they eventually reduced their area and left the exterior layer (deintercalation). Moreover, not all exterior cells that were in contact with the intercalating cell remained in contact with it (see green cells 2 and 4, Figure 7), and other cells that became in contact with it (red cells) did not maintain stable cell contacts. Overall, deintercalation events occurred frequently in morphant embryos but were not observed in control embryos, suggesting that the developmental basis for the epibolic arrest of PrP-1 morphant embryos is the impairment of morphogenetic cell movements directly controlled by E-cadherin. In agreement with this, the corresponding time-lapse videos show that PrP-1 morphant cells have a reduced ability to maintain tissue cohesion and to migrate in a coordinated fashion (compare Videos S1 and S2). Interestingly, and despite these defects, morphant cells seemed quite active in generating processes. To further clarify this issue, we made time-lapse recordings of single dissociated blastomeres (8 hpf), which show that their intrinsic motility is not affected by PrP-1 knockdown (compare Videos S3 and S4). To assess whether PrP-1 knockdown might affect other cellular events relying on proper cell–cell communication, we also controlled for changes in mitosis rates upon PrP-1 knockdown. Interestingly, we found a partial reduction in the numbers of dividing blastomeres in morphant embryos (Figure S3E); however, neither cell size nor overall cell density appeared to be significantly compromised in these embryos.

**Figure 8:**
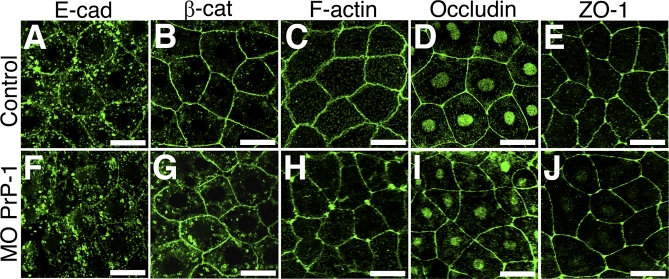
PrP-1 Regulation of Adherens, but Not Tight Junctions, in EVL Cells Differences in the subcellular distribution of various cell junction components between control and PrP-1 morphant embryos (MO) were evaluated in the polarized epithelial cells of the EVL at the shield stage (6 hpf). In control embryos, a marked membrane localization pattern can be seen for adherens junction (E-cadherin [E-cad], β-catenin [β-cat], and F-actin) (A–C) and tight junction markers (Occludin and ZO-1) (D and E). PrP-1 knockdown induces partial mislocalization of adherens junction components (F–H) but does not affect the distribution of classical tight junction markers (I and J). Scale bars indicate 20 μm.

It has previously been reported that mutation or down-regulation of E-cadherin specifically affects adhesion in the deep cell layer but not in the enveloping layer (EVL) of the zebrafish gastrula [23,24]. Similarly, the PrP-1 adhesion phenotype reported here appears restricted to deep cells, supporting the notion that PrP-1 modulates E-cadherin function, and that cell adhesion in the EVL may be controlled by additional mechanisms, such as the use of different types of cellular junctions. To further investigate this, we studied the distribution of markers for adherens and tight junctions in the EVL at 6 hpf. In PrP-1 morphants, EVL cells showed similar alterations in the distribution of E-cadherin, β-catenin, and F-actin as in deep cells (Figure 8A–8C and 8F–8H; for a *Z*-stack reconstruction of an embryo showing the size and morphology of both types of cells, see Video S5), indicating that PrP-1 regulates the stability of adherens junctions in both cell layers. In contrast, the membrane distribution of classical tight junction markers like occludin and ZO-1 remains unaffected in morphant EVL cells (Figure 8D, 8E, 8I, and 8J), whereas their presence in deep cells (control or morphant) could not be detected. Hence, the loss of PrP-1 and E-cadherin function may not significantly impair EVL cell adhesion due—at least in part—to its different cell junction composition.

### Adhesive and Signaling Properties of PrP

**Figure 9:**
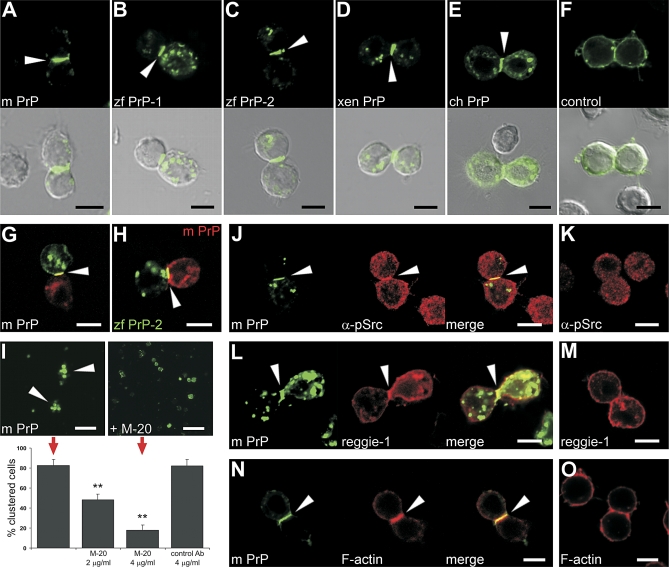
Cell Signaling and Adhesion in *Drosophila* S2 Cells upon PrP Expression (A–E) Expression of mouse PrP (m PrP [A]), zebrafish PrP-1 (zf PrP-1 [B]) and PrP-2 (zf PrP-2 [C]), *Xenopus* PrP (xen PrP [D]), and chick PrP (ch PrP [E]) EGFP fusion constructs in *Drosophila* nonadhesive S2 cells results in the induction of cell–cell contact formation and local PrP accumulation at cell contacts (white arrowheads). (F) Control EGFP fusion constructs do not induce this phenomenon (shown for m PrP at a fortuitous PrP-independent cell contact). (G) Cell contact formation and PrP accumulation (white arrowheads) in mixed S2 cell populations separately transfected with mouse EGFP- and DsRed-monomer-PrP constructs (G) exclude cell division artifacts. (H) The same result is obtained when mouse DsRed-monomer-PrP and zebrafish EGFP-PrP-2 constructs are used, suggesting PrP interaction across species. (I) Blocking of mouse PrP–mediated aggregation of S2 cells (red arrows, lower panel) by a polyclonal antibody against mouse PrP (M-20) shows that the formation of cell clusters (white arrowheads, upper left panel) is specifically induced by PrP. The effect was quantified as the number of cell contacts between S2 cells expressing mouse PrP in the absence (upper left) or presence (upper right) of M-20 or a control antibody at the concentrations indicated in the graph (double asterisks [**] indicate statistical significance at *p* < 0.01, one-way ANOVA test; error bars indicate SEM). (J–O) Strong anti–phospho-Src kinase immunostaining ([J] α-pSrc), as well as accumulation of rat reggie-1-DsRed-monomer ([L] reggie-1) and Alexa-568 Phalloidin ([N] F-actin) colocalize at mo PrP–mediated cell contacts (white arrowheads), but not at fortuitous PrP-independent cell contacts (K, M, and O). Scale bars in (A–H and J–O) indicate 5 μm; scale bar in (I) indicates 20 μm.

The formation of PrP-1-dependent small cell clusters in the absence of Ca^+2^ (Figure 5D) indicates that PrP might have its own adhesive properties. To clarify this, we employed nonadhesive *Drosophila* S2 cells, an experimental paradigm classically used to demonstrate the adhesive properties of membrane proteins [25]. Notably, S2 cells transfected with mouse and zebrafish EGFP- and DsRed-monomer-PrP constructs acquired the ability to aggregate and accumulate PrP at cell contacts (Figures 9A–9C and S4; Video S6). In contrast, control EGFP constructs did not accumulate even at fortuitous cell contacts (Figure 9F). Since S2 cells lack endogenous PrP [26] and do not express adhesion molecules, these experiments show that PrP expression leads to cell aggregation. Similarly, aggregation of cells transfected with frog and chicken PrP constructs indicates that the PrP-mediated adhesion is conserved across vertebrate classes (Figure 9D and 9E, arrowheads). To corroborate that PrP accumulates between distinct cells (as opposed to between dividing daughter cells), we reproduced these results using mixed S2 cell populations separately transfected with EGFP- and DsRed-monomer-PrP constructs (Figure 9G, arrowheads). In these experiments, untransfected cells did not form aggregates and remained excluded from the mouse PrP-transfected aggregates (data not shown), suggesting that cell aggregation is due to homophilic affinity between PrPs on apposing cell membranes. Interestingly, cross-interactions involving zebrafish and mouse PrPs also triggered aggregation of S2 cells (47 ± 1% aggregated cells compared to 83 ± 2% between mouse PrPs and 94 ± 2% between zebrafish PrP-2s, Figure 9H, arrowheads), revealing that, unlike the species restrictions that limit prion propagation [27], functional interactions can take place even between distantly related PrPs.

Our present work in zebrafish embryos revealed that PrP can regulate the function of E-cadherin; similarly, other studies have reported functional interactions between PrP and NCAM [10]. To exclude the possibility that PrP might cause S2 cell aggregation by indirectly activating these endogenous adhesion molecules, we immunostained S2 cells for DE-cadherin, DN-cadherin, and fasciclin II (*Drosophila* NCAM), and confirmed that these molecules are not re-expressed upon transfection with PrP constructs. To further test whether PrP itself can act as an adhesion molecule, we asked whether an anti-PrP antibody could interfere with the aggregation process. Indeed, clustering of cells transfected with mouse EGFP-PrP was drastically reduced by incubation with M-20, an anti-mouse PrP polyclonal antibody (Figure 9I, fluorescence images, arrowheads). The reduction in the percentage of aggregated cells was concentration-dependent, going from 83 ± 6% (control without antibody) to 48 ± 6% and 18 ± 5% in the presence of 2 and 4 μg/ml M-20, respectively. In contrast, incubation with 4 μg/ml of control antibody did not significantly affect cell clustering (82 ± 7%) (Figure 9I, histogram). Moreover, PrP-mediated aggregation of S2 cells was not affected by the presence of 0.5 mM EGTA (data not shown). Thus, we conclude that PrP can mediate homophilic cell adhesion in a Ca^+2^-independent manner.

Using T cells, we previously have shown that PrP can elicit signal transduction and reorganization of the actin cytoskeleton via Src-related tyrosine kinases, and that these events take place at specialized membrane microdomains defined by the presence of reggie/flotillin scaffolding proteins [14]. The present study shows that similar cellular signals between zebrafish blastomeres are inhibited upon PrP-1 knockdown (Figure 6T and 6U). Therefore, we investigated whether such signaling events are also induced in S2 cells upon the formation of Ca^+2^-independent, PrP-mediated contacts. In fact, activated Src-kinase (Figure 9J, arrowheads) and phosphotyrosine staining (Figure S5A–S5C), as well as coclustering of reggie/flotillin membrane microdomains (Figure 9L, arrowheads, and Figure S6A–S6E), and accumulation of F-actin (Figure 9N) could be seen to colocalize with PrP accumulation at cell contacts. Expression of control EGFP constructs did not induce such effects (Figure 9K, 9M, and 9O), strongly suggesting that the signaling observed is concomitant with PrP-mediated cell adhesion.

## Discussion

The present study shows that the loss of PrP function in a vertebrate can produce clear phenotypes amenable to cellular and molecular characterization. Our experiments reveal important roles of PrP during zebrafish development, PrP-1 regulating embryonic cell adhesion during gastrulation, and PrP-2 affecting later stages of neural development. Interestingly, the developmental expression and cellular localization patterns of PrP-2 suggest that it might play a role closer to that of mammalian PrP in the nervous system. In fact, mouse PrP is expressed throughout the developing nervous system in a pattern analogous to that of zebrafish PrP-2 [6]. Nevertheless, the differences in expression patterns and knockdown phenotypes between PrP-1 and −2 are most likely of transcriptional regulatory nature, because both proteins share important biological properties with mouse PrP, such as membrane anchorage, posttranslational processing, subcellular localization, and the ability to revert the PrP-1 knockdown phenotype. The strength of the PrP knockdown phenotypes in zebrafish is in sharp contrast with the lack of significant defects in PrP knockout mice. This striking difference may be due to the activation of gene compensatory mechanisms in the embryonic stem (ES) cells selected to derive the mouse knockouts [2]. For instance, a potential role of PrP in supporting axonal growth can be compensated by up-regulation of integrins in PrP knockout mice [11]. Hence, clear PrP phenotypes in mammals might become visible only upon replacement of the PrP gene with a dysfunctional (i.e., truncated) copy, such as in the “Shmerling phenotype” [28]. Examination of the immediate changes in gene expression upon PrP knockout in mammalian ES cells and embryos might help clarify this issue.

While PrP may establish interactions in *cis* or *trans* with adhesion molecules like NCAM [10], the specific accumulation of PrPs at the contacts between transfected N2a cells suggests that PrPs can also establish *trans*-interactions on apposing plasma membranes, as it has been hypothesized for brain endothelial cells [29]. In fact, our aggregation assays with zebrafish blastomeres and *Drosophila* S2 cells show that PrP itself can mediate Ca^+2^-independent homophilic cell adhesion, and that this adhesive property is conserved across vertebrate classes. Moreover, our results also demonstrate that PrP interactions play a regulatory role in vivo, by eliciting the signal transduction events necessary to modulate Ca^+2^-dependent cell adhesion in the zebrafish gastrula. In particular, we show that PrP-1–mediated signaling influences proper processing and/or trafficking of E-cadherin from storage vesicles to adherens junctions at the plasma membrane. This upstream regulatory role of PrP-1 over of E-cadherin is underscored by the remarkable similarities between the developmental roles of these two molecules. For instance, zebrafish E-cadherin is maternally expressed at adherens junctions and required to regulate embryonic cell adhesion of deep but not EVL cells; mutation or knockdown of E-cadherin induce epibolic arrest and disaggregation of blastodermal cells [20,22,24]. In this study, we further strengthened the functional connection between PrP-1 and E-cadherin, by showing that they interact genetically to control distinct cell morphogenetic movements required for zebrafish gastrulation.

Our experiments suggest that the regulation of E-cadherin by PrP-1 is likely to occur indirectly via signal transduction, and not through direct physical interaction. Accordingly, PrP colocalizes, but does not physically interact, with E-cadherin in cell junctions of human enterocytes [30]. Likewise, the different embryonic localization patterns of PrP-1 (in discrete membrane patches) and E-cadherin (along entire cell–cell contacts) argue against a necessary physical interaction between the two. Moreover, discrete localization in patches at cell contacts, and regulation of E-cadherin function via signaling have also been described for the wnt11 signaling pathway [31,32]. Interestingly, we find that PrP-1 knockdown also results in increased cytoplasmic accumulation of β-catenin. Since the loss of β-catenin signaling is known to increase neuronal apoptosis in Alzheimer's disease patients [33], it would be interesting to study whether β-catenin or wnt signaling are affected along with PrP function during prion-induced neurodegeneration.

Our previous work on T cells revealed that PrP-mediated signaling can trigger activation of Src-related kinases (such as Fyn and Lck) and elevation of intracellular Ca^+2^ levels, along with reorganization of the actin cytoskeleton [14]. Here, we show that similar signaling events are induced upon PrP accumulation at cell contacts. Interestingly, Src-related kinases are known to regulate cell adhesion via direct phosphorylation of p120 and β-catenins [34,35]. Moreover, two Src-related kinases, Fyn and Yes, are required for Ca^+2^ signaling and for regulation of the actin cytoskeleton during zebrafish epiboly [36,37]. Thus, PrP signaling may modulate embryonic cell adhesion and actin cytoskeleton dynamics through the activation of Src-related kinases and associated targets.

Analysis of the PrP-2 phenotype is beyond the focus of this study. However, ongoing studies in our lab (E. Málaga-Trillo and L. Luncz, unpublished data) indicate that PrP-2 plays a role in the proliferation and differentiation of developing neurons in vivo, similar to what has been shown for mouse embryonic cells [9]; the precise signaling pathways involved in these processes remain to be clarified.

Altogether, our experiments reveal evolutionarily conserved roles of PrP in the maintenance of Ca^+2^-dependent and Ca^+2^-independent embryonic cell adhesion. On one hand, we showed that PrP can directly mediate homophilic cell adhesion and signaling via Src-related kinases. On the other hand, we uncovered a functional link between the activity of PrP at cell contacts and the regulation of cadherin-mediated cell adhesion. Furthermore, we have demonstrated that these roles of PrP are required in vivo to regulate morphogenetic movements that drive early zebrafish development. The implications of these findings for mammalian prion biology await further elucidation; however, they open new avenues for the study of PrP function and prion-induced neurodegeneration across vertebrate models.

## Materials and Methods

### Molecular cloning and mRNA synthesis.

The leader, core, and GPI-anchor signal fragments (144, 1,677, and 120 bp for PrP-1; 219, 1,485, and 138 bp for PrP-2; 105, 660, and 87 bp for mouse PrP; 126, 447, and 75 bp for *Xenopus* PrP; and 129, 588, and 84 bp for chick PrP, respectively) containing the necessary restriction sites were generated by PCR and cloned into pCRII-TOPO as separate fragments, before subcloning into the end vectors. The corresponding EGFP-PrP constructs were engineered through conventional cloning procedures, by inserting leader cDNAs into the NheI/AgeI; and core and/or GPI-anchor cDNAs into the BglII/EcoRI sites of pEGFP-C1 (BD Biosciences Clontech). Zebrafish PrP N-glycosylation mutants were engineered by introducing point mutations (asparagine to glutamine) in residues 509 and/or 514 of PrP-1, and 438 and/or 443 of PrP-2. DsRed-monomer-PrP constructs were generated by replacing the EGFP ORF with the DsRed-monomer sequence (BD Biosciences Clontech). For *Drosophila* S2 cell transfection experiments, the EGFP- and DsRed-PrP constructs were subcloned into the XbaI/ApaI sites of the pAc5.1/V5-HisA vector (Invitrogen, provided by V. Katanaev). For morpholino rescue experiments, PrP ORF cDNAS were subcloned into the EcoRI site of pCS2+ [38] (provided by Z. Varga) and transcribed in vitro using the mMESSAGE mMACHINE SP6 kit (Ambion). For colocalization studies in S2 cells, rat reggie-1 and −2, and zebrafish reggie-2a were cloned into the EcoRI/BamHI sites of the pDsRed-Monomer-N1 (BD Biosciences Clontech), and further subcloned into the EcoRI/NotI sites of the pAc5.1/V5-HisA vector.

### Whole-mount RNA in situ hybridization.

Zebrafish developmental stages are indicated after Kimmel [39] and in hours postfertilization (hpf). Whole-mount in situ hybridization was performed as described in http://zfin.org/zf_info/zfbook/chapt9/9.8.html. The globular domain regions of zebrafish *PrP-1* and *PrP-2* were cloned in pCRII-TOPO (Invitrogen) and used as templates for the synthesis of RNA in situ hybridization probes with the DIG RNA Labeling Kit (Boehringer). Transcription patterns were visualized on an Axioplan 2 compound microscope (Carl Zeiss) using Nomarski optics, photographed with a Zeiss Color Axiocam, and further processed with Adobe Photoshop 8.0.

### Morpholino knockdowns and mRNA rescue.

The following morpholinos were purchased from Gene Tools and designed to target two independent sequences at the 5′ UTRs of each zebrafish *PrP*: MO-PrP1–1 (5′-TGA GCA GAG AGT GCT GCG GGA GAG A-3′), MO-PrP1–2 (5′-CGC TTC TTC AAC CTT TTT ATG GAC C-3′), MO-PrP2–1 (5′-CCA AGG GAC AAC AAT CGC CCA AGA G-3′), MO-PrP2–2 (5′-AGG ACT CGC TTA AAA CAG CCC GAA G-3′), Control (5′-CCT CTT ACC TCA GTT ACA ATT TAT A-3′), and MO-Cdh1, targeting the first 25 coding base pairs of zebrafish E-cadherin [20]. All microinjections were performed at early cleavage stages (one- to four-cell stage) using a manual micromanipulator (Narishige) coupled to a Transjector 5246 (Eppendorf) under a Stemi 2000 stereomicroscope (Zeiss). After running specificity and dose-dependency controls, morpholinos were injected at a concentration of 0.8 ng/nl in 1× Danieau buffer (58 mM NaCl, 0.7 mM KCl, 0.4 mM MgSO_4_, 0.6 mM Ca(NO_3_)_2_, 5.0 mM HEPES [pH 7.6]) and 0.125% Phenol Red (Sigma); both PrP-1 morpholinos produced the same phenotype (Table S1). For double-knockdown experiments, low doses (0.4 ng/nl) of PrP-1 morpholino, E-cad morpholino, or both morpholinos were microinjected; the numbers of embryos with arrested gastrulation were given as the percentage of total embryos treated, and statistically analyzed with an unpaired *t*-test (two-tailed distribution; average ± standard error of the mean [SEM]; *n* = 3). For rescue experiments, morpholinos at 1.6 ng/nl in 1× Danieau buffer were coinjected with capped mRNAs at 80 pg/nl at a 1:1 ratio in 0.05 M KCl and 0.125% Phenol Red; for overexpression experiments mRNAs were microinjected at or 40 pg/nl. At least 300 embryos were microinjected per experiment (5-nl injection volume) and kept in E3 medium (5 mM NaCl, 0.17 mM KCl, 0.33 mM CaCl_2_, 0.33 mM MgSO_4_) at 28 °C; quantitation of phenotypes was carried out for 200 embryos per experiment. Phenotypes were photographed with Zeiss color and black & white Axiocam cameras on an Axioplan 2 microscope using Nomarski optics. Images were further processed with Adobe Photoshop 8.0. Apoptotic cells in fixed embryos were stained with the TUNEL method using the In situ cell death detection kit, AP (Roche), images were acquired on a LUMAR.V12 (whole mounts) or Axioplan 2 (flat mounts) microscopes (Zeiss). Embryos treated with 10% ethanol for 5 min were used as positive apoptotic controls. In addition, embryos were stained with DAPI (100 ng/ml) at room temperature (RT) for 30 min, and examined for their nuclear morphology and the presence of apoptotic bodies.

### Time-lapse recordings.

To analyze gastrulation cell movements, control and morphant embryos at 75% epiboly (8 hpf) were mounted and recorded essentially as previously described [22,40], using an Axioplan 2 microscope under Nomarski optics. For analysis of single-cell behavior, isolated blastomeres were obtained as described below, mounted live in Ringer's solution and similarly recorded. Subsequently, the recordings were analyzed and converted to movies using Axiovision 4.6. To illustrate differences in radial intercalation, selected images from the time-lapse sequence were imported into Adobe Photoshop and pseudocolored to facilitate visualization.

### Cell culture and transfection.

N2a cells were maintained in 10% FCS MEM (Invitrogen), supplemented with l-glutamine, pyruvate, and penicillin-streptomycin at 37 °C and 5% CO_2_. Cells were grown on poly-lysine–coated coverslips for 24 h prior to transient transfection using Lipofectamine 2000 (Invitrogen). S2 cells were maintained in 10% FCS Schneider's Medium (AMIMED), supplemented with l-glutamine and penicillin-streptomycin at 24 °C. Cells were grown for 24 h prior to transient transfection using Effectene (QIAGEN). Analyses were performed 20 h (N2a) and 24 h (S2) after transfection.

### PI-PLC and PNGase F incubations.

To assay for functional GPI-anchoring, transiently transfected N2a cells were treated with PI-PLC (Roche) as previously described [41], and visualized by fluorescence microscopy. To determine glycosylation states, N2a cells were grown in six-well plates, transiently transfected, lysed, and incubated with N-Glycosidase F (Roche) as reported before [42]; samples were then analyzed by western blot using a monoclonal anti-GFP antibody (Roche). Additionally, N2a cells were transiently transfected with the zebrafish PrP N-glycosylation mutants, lysed, and analyzed by western blot using the anti-GFP antibody.

### Aggregation assays in *Drosophila* S2 and zebrafish embryonic cells.

S2 cells were transfected with mouse EGFP-PrP, DsRed-monomer-PrP, or zebrafish EGFP-PrP-2, and after 24 h, incubated in 0.05% trypsin in PBS for 5 min at RT. After washing, cells were resuspended in 10% FCS Schneider's Medium alone or supplemented with M-20 polyclonal anti-mouse PrP antibody (Santa Cruz Biotechnology), control antibody (mouse IgG; BD Biosciences), or EGTA at the concentrations indicated in the main text. After 2 h, cells were mounted for quantification. Three low-magnification fields of equal cell density were randomly taken from each experiment, and the cell clusters were scored (groups of three or more fluorescent cells). Cell contacts were quantified and given as the percentage of total transfected cells (average ± SEM; *n* = 3, ∼200 transfected cells per experiment; one-way ANOVA test).

Control zebrafish embryos, as well as embryos injected with lissamine-tagged PrP-1 morpholino or PrP-1 mRNA, were staged and collected in groups of approximately 50 individuals, dechorionated with pronase (2 mg/ml; Sigma) and mechanically dissociated to a single-cell suspension by pipetting for 5 min in Ringer's solution (116 mM NaCl, 2.9 mM KCl, and 5 mM HEPES [pH 7.2]) supplemented with 5 mM EDTA and 0.5 mM EGTA. The dissociated cells were collected by centrifugation, washed twice, and used for western blot analysis, or resuspended in Ringer's solution with or without 1.8 mM CaCl_2_ to test for Ca^+2^ dependence. Control, PrP-1 morphant cells, a 1:1 mixture of both, or PrP-1 overexpressing cells were then transferred to microfuge tubes, allowed to aggregate for various periods of time up to 45 min at 28 °C, and mounted for visualization and quantitative evaluation. The number of single cells and cells in aggregates were pooled and given as the percentage of total cells: approximately 200 cells were counted per experiment, eight independent experiments were considered (*n* = 8, average ± SEM), and statistically analyzed with a one-way ANOVA test. *Drosophila* S2 and zebrafish embryonic cells were imaged using Plan-NEOFLUAR 20× or 40× objectives and an AxioCam HRm on an Axioplan 2 microscope. Images were further processed with Corel PHOTO-PAINT 11.

### Cell transplantations.

Twenty to 30 cells from donor embryos labeled with lissamine-tagged morpholinos were transplanted into unlabeled host embryos essentially as described before [43] using the Transferman NK 2 and CellTram Vario micromanipulators (Eppendorf) on an Axiovert 200 microscope (Zeiss), and monitored on a LUMAR.V12 stereomicroscope before being fixed and mounted for observation. An unrelated morpholino that binds to the 5′ leader sequence of the pCS2+ vector was used as a specificity control.

### Antibody and F-actin stainings of cells and embryos.

N2a cells were grown on polylysine-coated coverslips and fixed for 15 min in 4% paraformaldehyde (PFA) 24 hours after transfection. S2 and zebrafish blastomeres cells were immobilized on Alcian blue–coated coverslips, fixed in 4% PFA for 15 min and mounted, or permeabilized with 0.1% Triton X-100 in PBS, probed for 1 h at RT with primary antibody or stained with 1:1,000 Alexa-488 or −568 Phalloidin (Molecular Probes), followed by incubation in 1:1,000 diluted Cy-3 or Alexa-488 conjugated goat anti-rabbit or donkey anti-mouse secondary antibodies (Jackson ImmunoResearch), also for 1 h at RT. The following primary antibodies were used: polyclonal anti-phospho-Src (Tyr416; Cell Signaling Technology) diluted 1:1,000, polyclonal anti-phosphotyrosine antibody (PY350; Santa Cruz Biotechnology) diluted 1:500, monoclonal anti-phosphotyrosine (P-Tyr-100; Cell Signaling Technology) diluted 1:500, prion monoclonal antibody (6H4; Prionics) diluted 1:1,000, and polyclonal anti-Fyn (FYN3; Santa Cruz Biotechnology) diluted 1:500. For analysis of PrP-1 and E-cadherin expression levels, ten control and morpholino embryos were dechorionated, deyolked, lysed, and analyzed by western blot using a purified mouse monoclonal anti–E-cadherin antibody (610182; BD Biosciences) diluted 1:2,000; a purified rabbit polyclonal anti–PrP-1 serum (generated in our lab) diluted 1:4,000; and a goat polyclonal IgG against γ-tubulin (C-20: sc-7396; Santa Cruz Biotechnology) diluted 1:200 as loading control. The anti–PrP-1 serum was not suited for immunofluorescence.

Zebrafish embryos were staged and fixed in 4% PFA overnight at 4 °C. After three washes in PBS-T (0.1% Triton in PBS) and a 1-h incubation in PBS-DT (1% DMSO in PBS-T), they were blocked for 4 h at RT in PBS-DT containing 10% goat serum, incubated with primary antibody (or stained with 1:100 Alexa-488 Phalloidin; Molecular Probes), washed three times in PBS-T, incubated with secondary antibody, and washed three more times in PBS-T. All washes were performed for 5 min at RT; antibody incubations were carried out overnight at 4 °C. The following primary antibodies were used: zebrafish cdh1 rabbit antiserum [20], purified mouse anti–E-cadherin (BD Biosciences), rabbit polyclonal anti–β-catenin (C2206; Sigma), rabbit polyclonal anti-phosphohistone H3[pSer10] (HO412; Sigma), and rabbit polyclonal Rab11 antibody (ab3612; Biozol) at 1:1,000 dilutions; Alexa-488 conjugated goat anti-rabbit secondary antibody at 1:1,000 (Jackson Immunoresearch). Quantification of E-cadherin/Rab11 colocalization was carried out on double-immunostained embryos. For each type of cell (EVL or deep cell [DC]), approximately 25 cells per embryo and approximately five control or morphant embryos were analyzed per experiment. The number of colocalizations per cell were pooled, and the results were statistically analyzed with an unpaired *t*-test (*n* = 3, two-tailed distribution). Quantification of immunostained mitotic cells/embryo was carried out on flat mounts of 15 control and 15 morphant embryos, and statistically analyzed with an unpaired *t*-test (two-tailed distribution; average ± SEM; *n* = 15). Visualization was carried out on Axioplan 2 and confocal LSM 510 laser-scanning microscopes (Zeiss). Images and fluorescence profiles were obtained with LSM 510 software (Zeiss) and further processed using Corel PHOTO-PAINT 11 and Adobe Photoshop 8.0.

## Supporting Information

Alternative Language Abstract S1 Translation of the Abstract into Spanish by Edward Málaga-Trillo (30 KB DOC)

Alternative Language Abstract S2 Translation of the Abstract into French by Houari Abdesselem (30 KB DOC)

Alternative Language Abstract S3 Translation of the Abstract into German by Aimilia Sempou (31 KB DOC)

Alternative Language Abstract S4 Translation of the Abstract into Greek by Aimilia Sempou (27 KB DOC)

Alternative Language Abstract S5 Translation of the Abstract into Portuguese by Alejandro Pinzón Olejua (29 KB DOC)

Alternative Language Abstract S6 Translation of the Abstract into Russian by Vsevolod Bodrikov (27 KB DOC)

Figure S1 PrP Overexpression Affects Zebrafish Gastrula and Neural Stages (A) Control embryos at the dome stage (4.3 hpf) are morphologically symmetrical and initiate epiboly in a uniform manner.
(B–D) Overexpression of zebrafish (zf) PrP-1 ([B] zf PrP-1), PrP-2 ([C] zf PrP-2), or mouse (m) PrP ([D] m PrP) results in earlier morphogenetic movements at one end of the blastodisc, causing a distinctive deformation of the embryo shape (∼60%, *n* = 200, arrowheads).
(E–L) At prim-5 (24 hpf) (E–H), and long pec (48 hpf) (I–L) stages, the normal development of the head in control embryos (E and I) contrasts sharply with the small brains and reduced, asymmetric (F–H and J–L), or even fused (K) eyes observed in embryos overexpressing zf PrP-1 (F and J), zf PrP-2 (G and K), or m PrP (H and L).
(M–O) The asymmetric epiboly phenotype (M)-seen here for overexpression of zf PrP-1 at 6 hpf- is not due to asymmetric distribution of mRNA, as lateral (N) and animal pole (O) fluorescence views of the same embryo show.
(A–D, M, and N) show lateral views; (E–L) rostral views; and (O) animal pole view.
(7.03 MB TIF)

Figure S2 Expression of Endogenous PrP, EGFP- and DsRed-Monomer–Tagged PrPs in N2a Cells (A) Local accumulation of zebrafish PrP-1 (zf PrP-1) at a cell–cell contact (white arrowhead) is observed only when both cells forming the contact express the PrP construct.
(B) Immunostaining using the 6H4 monoclonal antibody against mouse PrP shows the plasma membrane localization and accumulation at cell contacts of the endogenous PrP in N2a cells ([B], arrowhead and total fluorescence profile).
(C, E, and G) Mouse PrP (m PrP) and zebrafish PrP-2 (zf PrP-2) DsRed-monomer fusion proteins localize at the plasma membrane and show accumulation at cell contacts ([C and G], arrowheads and total fluorescence profiles); zebrafish DsRed-monomer-PrP-1 (zf PrP-1) localize almost exclusively at contacts ([E], arrowhead and total fluorescence profile).
(D, F, and H) Accumulation of PrP at cell contacts is lost in DsRed-monomer constructs containing only the leader and GPI-anchor signals of m PrP (D), zf PrP-1 (F), and zf PrP-2 (H).
(I and J) Zebrafish EGFP-PrP-1 and −2 ([I and J], left panels) localize at the plasma membrane via a GPI anchor, as evidenced by phosphatidylinositol-specific phospholipase C (PI-PLC) treatment ([I and J], right panels).
(K) Western blot analysis using an anti-GFP monoclonal antibody of cell extracts incubated in the presence (+) or absence (−) of PNGase F reveals that m PrP, zf PrP-1, and zf PrP-2 EGFP fusion proteins are N-glycosylated in N2a cells.
Scale bars in (A–J) indicate 10 μm.
(2.56 MB TIF)

Figure S3 Effect of PrP-1 Knockdown on Apoptosis and Mitosis Rates (A–D) DAPI stainings on 6-hpf embryos (B and D) do not reveal morphological signs of increased apoptosis (such as chromatin condensation and nuclear fragmentation) in detached knockdown cells (C) compared to control embryonic cells (A).
(E) PrP-1 morphant embryos show a clear reduction in the number of mitotic cells at 6 hpf (*p* = 4.3 × 10^−7^; triple asterisks [***] indicate statistical significance at *p* < 0.001); however, neither cell size nor overall cell density seems significantly affected (see main text).
Scale bars in (A–D) indicate 10 μm.
(9.82 MB TIF)

Figure S4 Adhesive Properties of PrPs in *Drosophila* S2 Cells Heterologous expression of PrPs induces cell aggregation, as shown for zebrafish PrP-2 (zf PrP-2) EGFP (A) and DsRed-monomer (B) fusion proteins. Accumulation at cell contacts of mouse PrP (m PrP, [B]), zf PrP-1 (C), and zf PrP-2 (D) DsRed-monomer fusion proteins are equally evident (arrowheads). Scale bars indicate 5 μm.
(6.57 MB TIF)

Figure S5 Accumulation of PrPs at Cell Contacts Correlates with Increased Levels of Tyrosine Phosphorylation in *Drosophila* S2 Cells (A–E) Accumulation of mouse DsRed-monomer-PrP (m PrP) and zebrafish EGFP- and DsRed-monomer-PrP-2 (zf PrP-2) at S2 cell contacts colocalizes with anti-phosphoSrc (α-pSrc, [A]) and anti-phosphotyrosine immunostaining (α-pTyr, [B–E], arrowheads), indicating recruitment and activation of Src tyrosine kinases.
(F) A control mouse DsRed monomer-PrP lacking the PrP core does not cause this effect.
Scale bars indicate 5 μm.
(8.00 MB TIF)

Figure S6 PrP-Mediated Cell Contacts Recruit Rat and Fish Reggie/Flotillin Proteins in *Drosophila* S2 Cells Accumulation of mouse EGFP-PrP (m PrP) and zebrafish EGFP-PrP-2 (zf PrP-2) at cell contacts colocalize with the recruitment of rat reggie-1 and -2 as well as zebrafish reggie-2a (zf reggie-2a) DsRed-monomer fusion proteins ([A–E], arrowheads). Scale bars indicate 5 μm.
(9.72 MB TIF)

Table S1 Effect of PrP-1 Knockdown and mRNA Rescue on Zebrafish Development (83 KB DOC)

Table S2 Effect of PrP-1 Knockdown and mRNA Rescue (Using EGFP Fusions) on Zebrafish Development (83 KB DOC)

Video S1 Cell Behavior during Epiboly in a Control Embryo Time-lapse recording of gastrulation cell movements in the epiblast at 8 hpf (lateral view). The coordinated migration of blastomeres and the general cell cohesion of the epiblast can be seen, along with some dividing cells. Radial intercalation events normally take place (see Figure 7), with cells from the interior cell layer entering the exterior cell layer and flattening out (building cell contacts with the surrounding cells). No instances of deintercalation could be observed. The recording was made over 20 min at four frames per minute. The images were converted into a video using the Axiovision 4.7 software (Zeiss).
(3.98 MB MOV)

Video S2 Cell Behavior during Epiboly in a PrP-1 Morphant Embryo Time-lapse recording of gastrulation cell movements in the epiblast at 8 hpf (lateral view). The coordinated migration of blastomeres is impaired and, in general, cell cohesion in the epiblast is severely disrupted, as more rounded cells are evident, with more and larger gaps between them. Around the gaps, cells can be seen actively extending protrusions and making unstable cell contacts. Radial intercalation events take place, but morphant cells do not show the ability to flatten out and integrate within the exterior layer (see Figure 7). The recording was made over 20 min at four frames per minute. The images were converted into a video using the Axiovision 4.7 software (Zeiss).
(5.07 MB MOV)

Video S3 Intrinsic Motility of a Single Blastomere from a Control Embryo Time-lapse recording of the behavior of a single blastomere at 8 hpf. Embryos were dissociated as described in Materials and Methods, and mounted in Ringer's solution for visualization. The normal rotatory movement and exploratory behavior of a single cell with a leading edge can be appreciated. The recording was made over 2 min at one frame per second. The images were converted into a video using the Axiovision 4.7 software (Zeiss).
(724 KB MOV)

Video S4 Intrinsic Motility of a Single Blastomere from a PrP-1 Morphant Embryo Time-lapse recording of the behavior of a single blastomere at 8 hpf. Embryos were dissociated as described in Materials and Methods, and mounted in Ringer's solution for visualization. The rotatory movement, exploratory behavior, and rotation speed of the cell appear normal compared to those of the control cell shown in Video S3. The recording was made over 2 min at one frame per second. The images were converted into a video using the Axiovision 4.7 software (Zeiss).
(762 KB MOV)

Video S5 Size and Morphology of Different Cell Types throughout a Zebrafish Gastrula A whole-mount F-actin–staining of a 6-hpf embryo using Alexa-488 Phalloidin was examined by confocal laser-scanning microscopy; 21 images from the animal pole were recorded to create a stack in the *z*-axis. The slices were converted into a video using the export options of the Zeiss LSM 510 software.
(8.03 MB MOV)

Video S6 Local Accumulation of PrP at a Cell Contact Three-dimensional (3D) reconstruction of PrP accumulation along an entire cell contact. S2 cells expressing mouse EGFP-PrP were examined by confocal laser-scanning microscopy; 19 images were recorded to create a stack in the *z*-axis. The full projection of a *Z*-stack was converted into a video using the 3D options of the Zeiss LSM 510 software.
(5.98 MB MOV)

## Abbreviations

EVL: enveloping layer
hpf: hours postfertilization
N2a: neuroblastoma 2a
PrP: prion protein
