# Supplementary material for: Regulation of Embryonic Cell Adhesion by the Prion Protein
Source: PLoS Biol. 2009 Mar 10;7(3):e1000055. doi: 10.1371/journal.pbio.1000055 (PMC2653553; doi:10.1371/journal.pbio.1000055)
Supplement: Supplementary file 1 [file pbio.1000055.sd001.doc]

**Regulación de la adhesion celular embrional por parte de la proteína de prion**

**(Traducido del inglés por Edward Málaga-Trillo)**

Si bien las proteínas de prión (PrPs) juegan un papel clave durante las enfermedades neurodegenerativas letales, su función fisiológica es pobremente comprendida, ya que los ratones knockout para PrP se desarrollan de manera relativamente normal. En este estudio reportamos un fuerte fenotipo de pérdida de función de PrP en embriones tempranos de pez cebra, caracterizado por la pérdida de adhesión celular y el arresto de la gastrulación. ARNms de PrP de pez cebra y de ratón pueden rescatar parcialmente este fenotipo de *knockdown*, lo cual implica que la función de PrP está conservada entre las especies. Utilizando células de pez cebra, ratón y mosca drosófila, demostramos que PrP: 1) efectúa adhesión celular homofílica Ca^+2^ independiente, así como señalización celular; y 2) modula la adhesión celular Ca^+2^ dependiente, a través de la regulación del tráfico de E-caderina hacia la membrana plasmática. Análisis de microscopía time-lapse *in vivo* revelan que el arresto en la gastrulación de embriones knockdown para PrP se produce debido a deficiencias en movimientos celulares morfogenéticos que requieren la adhesión mediada por E-caderina. Experimentos de transplantación celular indican además que la regulación de la adhesión celular por parte de la PrP es celular-autónoma. Más aun, hallamos que la acumulación local de PrP en sitios de contacto celular es concomitante con la activación de kinasas Src, el reclutamiento de microdominios reggie/flotillin, y la reorganización del citoesqueleto de actina. Todo esto concuerda con un papel de PrP en la modulación de la adhesión celular a través de señales intracelulares. En suma, nuestro trabajo revela roles evolutivamente conservados de PrP en comunicación celular, los cuales finalmente inciden sobre la estabilidad de las uniones intercelulares adherentes a lo largo del desarrollo embrionario.
