# Supplementary material for: Regulation of Embryonic Cell Adhesion by the Prion Protein
Source: PLoS Biol. 2009 Mar 10;7(3):e1000055. doi: 10.1371/journal.pbio.1000055 (PMC2653553; doi:10.1371/journal.pbio.1000055)
Supplement: Supplementary file 2 [file pbio.1000055.sd002.doc]

**Régulation embryonnaire de l’adhésion cellulaire par la protéine Prion**

**(Traduit de l’anglais par Houari Abdesselem)**

Les protéines Prion (PrPs) jouent un rôle clé dans des maladies neurodégénératives et pourtant leur fonction physiologique demeure jusqu’á présent inconnue, étant donné que les souris knockout pour la PrP se développement normalement. Dans cet article, on reporte sur un phénotype fort de perte de fonction de la PrP chez l’embryon de poisson zèbre, caractérisé par la perte de l’adhésion cellulaire et l’arrêt de la gastrulation. Les ARNms de la PrP de poisson zèbre ou de la souris peut partiellement sauver ce phénotype knockdown, ce qui indique que la fonction de PrP est conservée entre les espèces. En utilisant des cellules du poisson zèbre, de la souris, et de la mouche drosophile, on montre que la PrP: 1) intervient dans l’adhésion cellulaire homophilique Ca^+2^-indépendante et dans les signalisations cellulaires; et 2) module l’adhésion cellulaire Ca^+2^-dépendante en régulant la délivrance des E-[cadhérine](http://fr.wikipedia.org/wiki/Cadhérine)s á la membrane plasmique. Analyses de microscopie time lapse *in vivo* montrent que l’arrêt du développement embryonnaire chez les embryons knockdown pour la PrP est du a l’altération des mouvements cellulaires morphogéniques qui dépendent de l’adhésion cellulaire mediée par les E-[cadhérine](http://fr.wikipedia.org/wiki/Cadhérine)s. Des expériences de transplantation cellulaire indiquent aussi que la régulation embryonnaire de l’adhésion cellulaire par la PrP est autonome cellulaire. On outre, on trouve que l’accumulation de la PrP dans les sites de contact entre cellules est concomitante avec l’activation des kinases Src, le recrutement des micro-domaines reggie/flotillin, et la réorganisation du cytosquelette d’actine, ce qui est cohérent avec le rôle de la PrP dans la modulation de l’adhésion cellulaire via les signalisations intracellulaires. Ensemble, nos données révèlent des rôles évolutivement conservés de la PrP dans la communication cellulaire, ayant un effet sur la stabilité des jonctions intercellulaires adhérents au cours du développement embryonnaire.
