# Supplementary material for: Regulation of Embryonic Cell Adhesion by the Prion Protein
Source: PLoS Biol. 2009 Mar 10;7(3):e1000055. doi: 10.1371/journal.pbio.1000055 (PMC2653553; doi:10.1371/journal.pbio.1000055)
Supplement: Supplementary file 3 [file pbio.1000055.sd003.doc]

**Regulation der embryonalen Zelladhäsion durch das Prion Protein**

**(Übersetzt aus dem Englischen von Aimilia Sempou)**

Prion Proteine (PrPs) sind Schlüsselspieler in fatalen neurodegenerativen Krankheiten, jedoch bleibt ihre physiologische Funktion ungeklärt, da PrP knockout-Mäuse weitgehend normal erscheinen. Hier berichten wir über einen starken PrP “loss-of-function”-Phänotyp im frühen Zebrafisch-Embryo, der sich durch den Verlust der embryonalen Zelladhäsion und die Arretierung der Gastrulation auszeichnet. Zebrafisch- und Maus- PrP-mRNAs können diesen knockdown Phänotyp zum Teil rückgängig machen, was auf eine evolutiv konservierte Funktion des Proteins hindeutet. Durch die Verwendung von Zebrafisch-, Maus- und Drosophila-Zellen wird hier gezeigt, dass PrP: 1) Ca^+2^-unabhängige homophile Zelladhäsion und Signaltransduktion vermittelt; und 2) Ca^+2^-abhängige Zelladhäsion moduliert, indem es den Transport von E-Cadherin an die Zellmembran reguliert. *In vivo* time-lapse Analysen lassen erkennen, dass die für PrP-knockdown Embryonen charakteristische Arretierung der Gastrulation eine Folge von beeinträchtigten morphogenetischen Zell-Bewegungen ist, welche von der E-Cadherin vermittelte Adhäsion abhängig sind. Zelltransplantationsexperimente offenbaren, dass die Regulation der Embryonalen Zelladhäsion durch PrP zellautonom ist. Weiterhin finden wir, dass die lokale Akkumulation von PrP an Zell-Zell-Kontaktstellen mit der Aktivierung von Src-verwandten Kinasen, der Rekrutierung von Reggie/Flotillin-Mikrodomänen, und der Reorganisierung des Aktin-Zytoskeletts einhergeht, was im Einklang mit der Rolle von PrP in der Regulation von Zelladhäsion durch Signaling steht. Insgesamt enthüllen unsere Daten evolutiv konservierte Rollen von PrP in Zellkommunikation, die sich letztendlich auf die Stabilitat von Adherens Junctions während der Embryonalentwicklung auswirken.
