# Supplementary material for: Regulation of Embryonic Cell Adhesion by the Prion Protein
Source: PLoS Biol. 2009 Mar 10;7(3):e1000055. doi: 10.1371/journal.pbio.1000055 (PMC2653553; doi:10.1371/journal.pbio.1000055)
Supplement: Supplementary file 4 [file pbio.1000055.sd004.doc]

**Η πρωτείνη prion ως ρυθμιστικός παράγοντας της εμβρυϊκής διακυτταρικής προσκόλλησης**

**(Μετάφραση από τα Αγγλικά από την Αιμιλία Σέμπου)**

Οι πρωτεΐνες prion (PrPs) είναι παίκτες κλειδιά σε θανάσιμα νευροεκφυλιστικά νοσήματα. Η φυσιολογική λειτουργία τους παραμένει ωστόσο άγνωστη, αφού PrP knockout ποντίκια δεν παρουσιάζουν ιδιαίτερο φαινότυπο. Εδώ, αναφέρουμε έναν ισχυρό φαινότυπο σε πρώιμο εμβρυϊκό στάδιο του zebrafish, οφειλόμενο σε απώλεια της λειτουργίας της PrP. Χαρακτηριστικά του είναι η απώλεια της διακυτταρικής προσκόλλησης στο έμβρυο και η μη ολοκλήρωση σχηματισμού του γαστριδίου. PrP mRNAs από zebrafish ή ποντίκι μπορούν να επαναφέρουν το φυσιολογιικό φαινότυπο μερικώς, πράγμα που υποδηλώνει διατήρηση της λειτουργίας της PrP κατά την εξέλιξη. Χρησιμοποιώντας κυττάρα από zebrafish, ποντίκι και *Drosophila,* δείχνουμε πως η PrP: 1) συμμετέχει στην ανεξάρτητη από Ca^+2^, ομοφιλική κυτταρική προσκόλληση και τη σχετική σηματοδοτική οδό και 2) ρυθμίζει την εξαρτώμενη από Ca^+2^ κυτταρική προσκόλληση με το να ελέγχει την παραλαβή της Ε-Cadherin στην κυτταρική μεμβράνη. *In vivo* αναλύσεις time-lapse φανερώνουν ότι η αναχαίτιση σχηματισμού του γαστριδίου σε έμβρυα zebrafish με PrP knockdown οφείλεται στην αποτυχία των κυττάρων να εκτελέσουν κινήσεις που είναι μορφογόνες για το έμβρυο και που εξαρτώνται από διακυτταριικές επαφές μέσω E-Cadherin. Πειράματα μεταμόσχευσης κυττάρων αποκαλύπτουν ότι η ιδιότητα της PrP να ελέγχει την εμβρυϊκή διακυτταρική προσκόλληση είναι αυτόνομη σε κάθε κύτταρο. Επιπλέον, διαπιστώνουμε ότι η τοπική συγκέντρωση μορίων PrP στα σημεία επαφής κυττάρων συνοδεύεται από ενεργοποίηση κινάσεων τύπου Src, στρατολόγηση Reggie/Flotillin microdomains καθώς και αναδόμηση του κυτταροσκελετού ακτίνης, γεγονός που υποδεικνύει σηματοδότηση σχετική με τη λειτουργία της PrP στην διακυτταρική προσκόλληση. Συμπερασματικά, τα δεδομένα μας αναδεικνύουν λειτουργιίες της PrP, διατηρημένες στην εξέλιξη των ειδών, που αφορούν την διακυτταρική επικοινωνία και είναι καθοριστικές για το σχηματισμό των adherens junctions κατά την εμβρυϊκή ανάπτυξη.
