# Supplementary material for: Regulation of Embryonic Cell Adhesion by the Prion Protein
Source: PLoS Biol. 2009 Mar 10;7(3):e1000055. doi: 10.1371/journal.pbio.1000055 (PMC2653553; doi:10.1371/journal.pbio.1000055)
Supplement: Supplementary file 5 [file pbio.1000055.sd005.doc]

**Regulação da adesão celular embrionária pelas proteínas do prião**

**(Traduzido do Inglês por Alejandro Pinzón Olejua)**

Embora as proteínas do Prião (PrPs) desempenhem um papel chave em doenças neurodegenerativas fatais, a sua função fisiológica ainda é pouco compreendida, uma vez que ratos knock-out para PrP se desenvolvem de maneira relativamente normal. Neste estudo reportamos um fenótipo acentuado de perda de função de PrP em embriões de peixe-zebra, caracterizado pela perda de adesão celular e detenção da gastrulação. ARNms de PrP de peixe-zebra e rato podem resgatar parcialmente este fenótipo de knockdown, indicando desta forma uma conservação funcional da PrP. Através da utilização de células de peixe-zebra, rato e *Drosophila*, foi demonstrado que a PrP: 1) efectua adesão celular homofílica independente do Ca^+2^, como também sinalização celular e 2) modula a adesão celular dependente do Ca^+2^, mediante o controlo do tráfego de E-cadherinas à membrana plasmática. Análises de microscopia time-lapse *in vivo* revelam que a detenção da gastrulação em embriões com knock-down para PrP se deve a deficiências nos movimentos celulares morfogenéticos dependentes da adesão mediada por E-cadherina. Experiências de transplantação celular indicam ainda que a regulação da adesão celular por parte de PrP é celular-autónoma. Para além disso verificamos que a acumulação de PrP em locais de contacto celular é concomitante com a activação de quinases da família Src, o recrutamento de microdomínios de reggie/flotillin, e a reorganização do citoesqueleto de actina. Todas estas observações estão de acordo com um papel de PrP na modulação da adesão celular através de sinais intracelulares. A todo, os nossos dados revelam papéis evolutivamente conservados de PrP na comunicação celular, que incidem finalmente na estabilidade das uniões intercelulares aderentes ao longo do desenvolvimento embrionário.
