# Supplementary material for: Regulation of Embryonic Cell Adhesion by the Prion Protein
Source: PLoS Biol. 2009 Mar 10;7(3):e1000055. doi: 10.1371/journal.pbio.1000055 (PMC2653553; doi:10.1371/journal.pbio.1000055)
Supplement: Supplementary file 6 [file pbio.1000055.sd006.doc]

**Роль белка Прион (PrP) в регулировании адгезии эмбриональных клеток.**

**(Переведено с Английского на Русский язык Всеволодом Бодриковым)**

Прионовые белки (PrPs) являются ключевыми молекулами в развитии летальных нейродегенеративных болезней, однако их физиологическая функция остаётся неясной, так как PrP нокаутные мыши имеют нормальный фенотип. Мы определили, что отсутствие белка PrP в эмбрионах зебрафиш (*Danio rerio*, zebrafish) приводит к потере адгезивных свойств эмбриональными клетками и остановке гаструляции. мРНК PrP мыши и зебрафиш способны частично компенсировать подобный фенотип вызванный снижением уровня PrP, что служит признаком функционального консерватизма белка PrP в процессе эволюции. Используя клетки зебрафиш, мыши и дрозофил (*Drosophila melanogaster*) нами было показано: 1) PrP является медиатором Ca^+2^-независимой клеточной адгезии и внутриклеточных сигнальных каскадов; и 2) PrP контролирует Ca^+2^-зависимую клеточную адгезию путём регулирования доставки белка Е-кадгерина в плазматическую мембрану. Используя *in vivo* time-lapse анализ мы показали, что дефектный фенотип эмбрионов вызванный снижением уровня белка PrP образуется в следствии нарушения морфогенетического движения, которое основывается на Е-кадгерин зависимой адгезии. Эксперименты с применением трансплантации клеток выявили, что PrP зависимая регуляция эмбриональной клеточной адгезии обладает клеточной автономией. Так же мы обнаружили, что аккумуляция белка PrP в местах клеточных контактов сопровождается активацией нерецепторных киназ семейства Src, перераспределением микродоменов reggie/flotillin, и реорганизацией актиновых компонентов цитоскелета, что подтверждает роль белка PrP в контроле клеточной адгезии путем регулирования внутриклеточных сигнальных каскадов. Как видно из представленных данных, нами была выявлена эволюционно консервативная роль белка PrP в клеточном взаимодействии, заключающаяся во влиянии на стабильность адгезионных соединений на протяжении эмбрионального развития.
