# Supplementary material for: Regulation of Embryonic Cell Adhesion by the Prion Protein
Source: PLoS Biol. 2009 Mar 10;7(3):e1000055. doi: 10.1371/journal.pbio.1000055 (PMC2653553; doi:10.1371/journal.pbio.1000055)
Supplement: Supplementary file 7 [file pbio.1000055.st001.doc]

**Table S1**. Effect of PrP-1 knockdown and mRNA rescue on zebrafish development

|  | Control | MO-PrP1-1 | MO-PrP1-2 | PrP-1 rescue | PrP-2 rescue | mPrP rescue |
| --- | --- | --- | --- | --- | --- | --- |
| Arrested embryos (at 9 hpf) | 10 (5%) | 189 (94.5%) | 181 (90.5%) | 42 (21%) | 76 (38%) | 94 (47%) |
| Degree of epiboly (at 9 hpf) | ~90% | ~50% | ~50% | ~90% | ~90% | ~70% |
| Dead embryos (at 24 hpf) | 10 (5%) | 184 (92%) | 175 (87.5%) | 40 (20%) | 73 (36.5%) | 89 (44.5%) |

Variation in the numbers of arrested embryos, degree of epiboly, and mortality rates between control embryos, PrP-1 morphant embryos (MO-PrP1-1 and MO-PrP1-2), and embryos rescued by coinjection of MO-PrP1-1 with zebrafish PrP-1 (PrP-1 rescue), PrP-2 (PrP-2 rescue) or mouse PrP (mPrP rescue) mRNAs. n=200 injected embryos were analyzed per experiment.
