# Supplementary material for: Regulation of Embryonic Cell Adhesion by the Prion Protein
Source: PLoS Biol. 2009 Mar 10;7(3):e1000055. doi: 10.1371/journal.pbio.1000055 (PMC2653553; doi:10.1371/journal.pbio.1000055)
Supplement: Supplementary file 8 [file pbio.1000055.st002.doc]

**Table S2**. Effect of PrP-1 knockdown and mRNA rescue (using EGFP fusions) on zebrafish development

|  | Control | MO-PrP1-1 | EGFP-PrP1 rescue | EGFP-PrP-2 rescue | EGFP-PrPm rescue | EGFP control rescue |
| --- | --- | --- | --- | --- | --- | --- |
| Arrested embryos (at 9 hpf) | 6 (3%) | 183 (91.5%) | 45 (22.5%) | 80 (40%) | 102 (51%) | 175 (87.5%) |
| Degree of epiboly (at 9 hpf) | ~90% | ~50% | ~90% | ~90% | ~70% | ~50% |
| Dead embryos (at 24 hpf) | 4 (2%) | 172 (86%) | 41 (20.5%) | 68 (34%) | 105 (52.5%) | 174 (87%) |

Variation in mortality and epiboly rates between control embryos, PrP-1 morphant embryos (MO PrP-1), and embryos rescued by coinjection of MO-PrP1 with zebrafish PrP-1 (EGFP-PrP-1 rescue), PrP-2 (EGFP-PrP-2 rescue), mouse PrP (EGFP-mPrP rescue) or PrP-1 Leader-GPI anchor (EGFP-control rescue) EGFP fusion mRNAs. n=200 injected embryos were analyzed per experiment.
